# The Choice of Regimens Based on Bortezomib for Patients with Newly Diagnosed Multiple Myeloma

**DOI:** 10.1371/journal.pone.0099174

**Published:** 2014-06-11

**Authors:** Jingsong He, Li Yang, Xiaoyan Han, Gaofeng Zheng, Weiyan Zheng, Guoqing Wei, Wenjun Wu, Xiujin Ye, Jimin Shi, Wanzhuo Xie, Li Li, Jie Zhang, Weijia Huang, Yi Zhao, He Huang, Xuejin Zhang, Jiaping Fu, Zhen Cai

**Affiliations:** 1 The Bone Marrow Transplantation center & Multiple Myeloma Treatment Center, The First Affiliated Hospital of Medical College, Zhejiang University, Hangzhou, Zhejiang, P. R. China; 2 Department of Hematology, Red Cross Hospital in Hangzhou, Zhejiang, P. R. China; 3 Department of Hematology, Shaoxing People’s Hospital, Zhejiang, P. R. China; Moffitt Cancer Center, United States of America

## Abstract

**Introduction:**

Bortezomib has significantly improved multiple myeloma (MM) response rates, but strategies for choosing bortezomib-based regimens for initial MM therapy are not standardized. Here, we describe four bortezomib-based therapies in Chinese MM patients to determine the optimal chemotherapeutic approach.

**Methods:**

Newly diagnosed symptomatic MM patients at three hematological centers between February 1, 2006 and May 31, 2013 were treated with therapies including bortezomib plus dexamethasone (PD) or combinations of PD with either adriamycin (PAD), cyclophosphamide (PCD) or thalidomide (PTD) for every 28 days.

**Results:**

The overall response rate of all the 215 eligible patients was 90.2%. The ORR for PCD, PAD, PTD and PD were 97.4%, 93.2%, 85.3% and 77.8% while the effects with VGPR or better were 63.7%, 62.7%, 44.2% and 37.8%, respectively. The effect of ORR, VGPR and CR/nCR for the PCD regimen was better than the PD protocol. Median PFS for all patients was 29.0 months with significant differences observed among treatment groups. Median OS of all the patients was not reached, but three-drug combinations were superior to PD alone. Frequently observed toxicities were neutropenia, thrombocytopenia, fatigue, infection, herpes zoster, and peripheral neuropathy. The incidence of peripheral neuropathy (PN) in PTD group was significantly higher than other three groups, especially grade 2–3 PN. Treatment with anti-viral agent acyclovir significantly reduced the incidence of herpes zoster.

**Conclusions:**

Our experience indicated that bortezomib-based regimens were effective and well-tolerated in the Chinese population studied; three-drug combinations PCD, PAD were superior to PD, especially with respect to PCD.

## Introduction

Multiple myeloma (MM) is a malignant neoplasm of bone marrow plasma cells, representing the second most common hematologic malignancy worldwide [1]. Median survival for MM has historically been approximately 3–5 years with 5–8% of patients experiencing complete remission (CR) upon treatment with conventional therapy, such as melphalan and prednisone (MP) or vincristine, doxorubicin, dexamethasone (VAD), prior to the advent of novel therapies [1]–[3]. Bortezomib, a reversible inhibitor of the 26S proteasome, has anti-tumor activity conferred by multiple mechanisms. Clinical studies suggest that bortezomib is effective for the treatment of MM and offers improved remission and better survival [3]–[7]. At present there are bortezomib based combination chemotherapy with 2 or 3 drugs, but there is no clear strategy for choosing a regimen for different patients since few clinical trials are supported. Thus, we retrospectively analyzed the efficacy and adverse effects experienced by MM patients who received combination therapy based on bortezomib as the first-line therapy from three hematological centers in China and we report our findings here.

## Materials and Methods

### Patients

The study protocol was approved by the research ethics Committee of the First Affiliated Hospital, College of Medicine, Zhejiang University. All patients provided written informed consent and received bortezomib-based combination chemotherapy as first-line treatment. Patients with newly diagnosed symptomatic MM between February 1, 2006 and March 31, 2013 in three hematological centers who displayed either measurable blood or urine monoclonal protein (M protein) were included in this study. The diagnostic criteria were primarily derived from the World Health Organization (WHO) and we used both the International Staging System (ISS) and the Durie-Salmon (DS) staging for assessing patients.

### Treatment

Patients received bortezomib-based combination chemotherapy in 28-day cycles as follows: bortezomib plus dexamethasone (PD) or three-drug combinations of PD with Adriamycin (PAD), cyclophosphamide (PCD) or thalidomide (PTD). Among the above regimens, bortezomib (1.3 mg/m^2^) was given intravenously on days 1, 4, 8, 11, whereas dexamethasone (20 mg^/^day) was given intravenously on days 1–2, 4–5, 8–9, 11–12, adriamycin (10 mg/m^2^) was given intravenously on days 1–4, cyclophosphamide (200 mg/m^2^) was given intravenously on days 1–4 and thalidomide (100 mg) was administered orally each day.

Twenty patients received autologous stem cell transplantation (ASCT) after induction therapy including 3 cases who received PTD induction therapy, 4 cases who received PD, 5 cases who received PCD and 5 cases who received PAD. After induction therapy or ASCT, patients were given thalidomide (100 mg/d) for maintenance treatment until progression was observed or until they could not tolerate treatment due to adverse reactions.

Acyclovir was gived to 7 patients in the PTD group and to patients in the other groups. Routine anti-coagulation or anti-thrombotic agents were not used.

### Response and Adverse Events Assessment

The International Melanoma Working Group (IMWG) uniform response criteria were used for the evaluation of response, including CR, very good partial response (VGPR), partial response (PR), stable disease (SD) and progressive disease [8]. Progression-free survival (PFS) was calculated from the time of first treatment to the time of progressive disease or until the time of the last follow-up or until a patient death. Overall survival (OS) was calculated from the time of start of therapy initiation until the date of death from any cause or to the time of the last follow-up. Adverse events were assessed at each visit and graded according to the National Cancer Institute’s Common Terminology Criteria (version 3.0; NCI-CTC 3.0).

### Statistical Analysis

All patients were followed-up until June 30, 2013, and patient assessment of patients began after one course of chemotherapy. Clinical characteristics, adverse effects rates and the 3-year OS and PFS rates were analyzed by the Pearson *χ* ^2^ test. Response rates were compared with multivariate logistic regression analysis, odds ratios (ORs) and 95% confidence intervals (CIs) were estimated by logistic regression. Kaplan-Meier methods were used to generate survival distribution graphs and differences in survival (e.g., PFS and OS) and statistically compared by the log-rank test. Multivariate analyses of prognostic factors were performed using Cox regression modeling. All probability values were two-sided and A P-value of 0.05 indicated statistical significance. All the data can be found in the Supporting Information files of the submission.

## Results

### Patient Characteristics

Two hundred and fifteen patients with MM were eligible for inclusion in this retrospective analysis, including a total of 135 male patients and 80 female patients, and the male-to-female ratio was 1.7∶1. The median age at diagnosis was 60 years (range, 31–83 years). Of all the patients, 79.0% (170/215) were in stage III by DS staging while 76.3% (164/215) were in stage II and III by ISS staging. Baseline patient characteristics were well-balanced. The baseline demographic and disease characteristics of patients in deferent treatment groups were listed in Table 1.

**Table 1:** Patient characteristics and baseline demographics.

	Total	PCD	PAD	PDT	PD
Variable	(n = 215)	(n = 77)	(n = 59)	(n = 34)	(n = 45)
Age, n(%)					
<65	152(70.7)	53(68.8)	50(84.7)	24(70.6)	25(55.6)
≥65	63(29.3)	24(31.2)	9(15.3)	10(29.4)	20(44.4)
Gender, n(%)					
Male	135(62.8)	35(45.5)	45(76.3)	24(70.6)	31(68.9)
Female	80(37.2)	32(41.6)	24(40.7)	10(29.4)	14(31.1)
Type of myeloma, n(%)					
IgA	59(27.4)	23(29.8)	17(28.8)	7(20.6)	12(26.8)
IgG	101(47.0)	33(42.9)	26(44.1)	22(64.7)	20(44.4)
IgD	4(1.9)	0(0.0)	1(1.7)	1(2.9)	2(4.4)
Light chain	50(23.3)	21(27.3)	14(23.7)	4(11.8)	11(24.4)
Biphenotypic (IgG, IgA)	1(0.5)	0(0.0)	1(1.7)	0(0.0)	0(0.0)
Durie-Salmon Staging, n(%)					
1A	10(4.7)	2(2.6)	3(5.1)	2(5.9)	3(6.7)
2A	35(16.3)	10(13.0)	9(15.3)	5(14.7)	11(24.4)
3A	117(54.3)	47(61.0)	34(57.6)	17(50.0)	19(42.2)
3B	53(24.7)	18(23.4)	13(22.0)	10(29.4)	12(26.7)
International Staging System Staging, n(%)					
1	51(23.7)	20(26.0)	13(22.0)	4(11.8)	14(31.1)
2	79(36.7)	28(36.3)	23(39.0)	16(47.0)	12(26.7)
3	85(39.6)	29(37.7)	23(39.0)	14(41.2)	19(42.2)

### Number of Cycles and Responses

A total of 215 patients received 1 to 8 cycles of treatment with median of 3 cycles, including 36 cases only receiving 1 cycle, 41 cases receiving 2 cycles, 55 cases receiving 3 cycles, 83 cases receiving 4 or more cycles of chemotherapy. Response rates were listed in Table 2.

**Table 2:** Overall response rates of regimens.

	ORR			PR			VGPR			nCR/CR		
	N (%)	OR, 95%CI	*P*	N (%)	OR, 95%CI	*P*	N (%)	OR, 95%CI	*P*	N,%	OR, 95%CI	*P*
PD	35(77.8)	1^a^		18(40.0)	1		8(17.8)	1^b^		9(20.0)	1^c^	
PCD	75(97.4)	9.11, 1.75–47.32	0.009	26(33.8)	6.79, 1.23–37.36	0.028	21 (27.3)	10.49, 1.70–64.78	0.011	28(36.4)	13.19, 2.20–79.22	0.005
PAD	55(93.2)	4.64, 1.10–19.60	0.037	18(30.5)	3.17, 0.69–14.55	0.139	17(28.8)	5.57, 1.11–29.86	0.037	20(33.9)	7.15, 1.41–36.16	0.017
PDT	29(85.3)	1.62, 0.44–5.96	0.466	14(41.2)	1.50, 0.38–6.03	0.566	11(32.4)	2.65, 0.56–12.44	0.218	4(11.8)	0.95, 0.17–5.25	0.950

a Patients received PCD or PAD demonstrated significant higher ORR compared to PD (97.4%, 93.2% vs 77.8%, *P* = 0.009, 0.037).

b Response rates defined as VGPR for PCD and PAD were significantly higher than PD (27.3%, 28.8%, vs 17.8%, *P* = 0.011, 0.037).

c Rates of patients received CR/nCR in PCD and PAD were higher than PD (36.4%, 33.9%, vs 20.0%, *P* = 0.005, 0.017).

Overall response rate (≥PR, ORR) of all the 215 eligible patients was 90.2% including 26.5% VGPR and 28.4% CR/nCR. Table 2 depicts details about ORR and treatment groups.

The patients received PCD or PAD demonstrated significant higher ORR compared to PD (97.4%, 93.2% vs 77.8%, *P* = 0.009, 0.037). The response rate defined as VGPR or better in the all patients was 54.9%. Response rates defined as VGPR for PCD and PAD were significantly higher than PD (27.3%, 28.8%, vs 17.8%, *P* = 0.011, 0.037). Rates of patients received CR/nCR in PCD and PAD were higher than PD (36.4%, 33.9%, vs 20.0%, *P* = 0.005, 0.017). Patients receiving bortezomib-based therapies had a rapid treatment response with respect to ORR (77.2%) after one cycle, especially for the PCD group which performed better than other regimens and was superior to PD (especially for better than VGPR). Response for four regimens after every cycle are depicted in Table S1.

### PFS and OS

**Figure 1:**
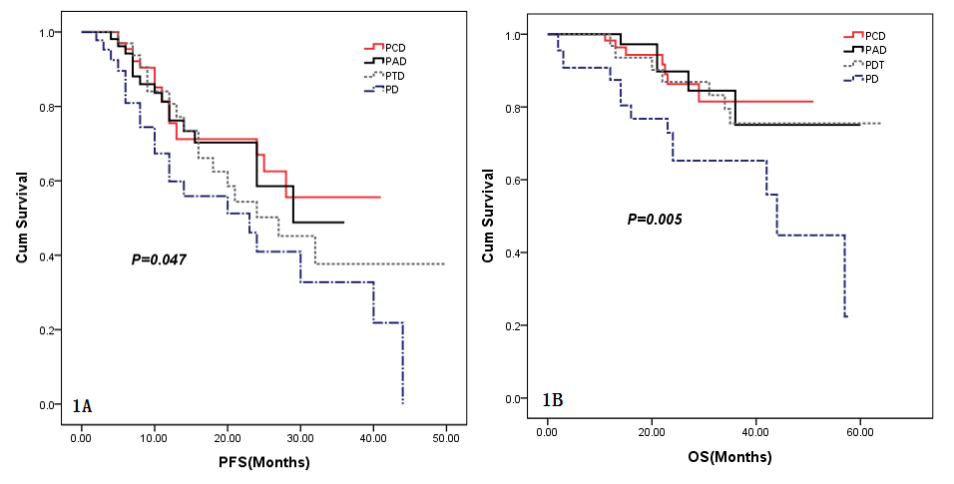
Kaplan-Meier survival curves for patients who received PCD, PAD, PTD and PD. **1A:** Median PFS of the 215 patients was 29.0 months (95% CI: 21.6–36.4 months). Median PFS was 27.0 months (95% CI: 15.9–38.1 months) for patients who received PDT, and 23.0 months (95% CI: 10.1–35.9 months) in PD respectively whereas the median PFS for PCD and PAD were not reached but with significant differences were observed among the groups (P = 0.047). The respective 3-year PFS was 71.4±6.1%, 70.3±7.1%, 50.2±9.7%, 46.1±9.8% with PCD, PAD, PTD and PD regimens, respectively. **1B:** Median OS of the 215 patients was not reached at 64 months in either treatment arm and significant differences were observed among the groups (P = 0.005; Fig. 1B). Median OS for the PD arm was 44.0 months (95% CI: 39.2–48.8 months) and this was not reached in the other treatment arms. Median OS for PTD, PCD and PAD was significantly longer than the PD group (P = 0.015, 0.013, 0.023). The respective 3-year OS was 86.3±5.3%, 75.1±11.0%, 75.5±8.1%, 65.3±8.8% for the PCD, PAD, PTD and PD regimens.

The median duration of follow-up for the 215 patients from diagnosis was 22.5 months (range, 2.0–64.0 months). The median PFS of the 215 patients was 29.0 months (95% CI: 21.6–36.4 months). The median PFS was 27.0 months (95% CI: 15.9–38.1 months) for patients who received PDT, and 23.0 months (95% CI: 10.1–35.9 months) for those in the PD group and median PFS for PCD and PAD groups were not reached. There were significant differences among the groups and with respect to multivariate analysis (Fig. 1A, Table 3). Respective 3-year PFS was 71.4±6.1%, 70.3±7.1%, 50.2±9.7%, 46.1±9.8% in PCD, PAD, PTD and PD regimens. The 3-year PFS rate of PCD was significantly higher than PTD and PD (*χ^2^* = 4.752, 7.414, *P* = 0.029, 0.006), but without statistical significance compared to PAD (*χ^2^* = 0.060, *P* = 0.806). The 3-year PFS rate of PAD was significantly higher than PD (*χ^2^* = 5.524, *P* = 0.019), but without statistical significance compared to PTD (*χ^2^* = 3.491, *P* = 0.062). The 3-year PFS rate of PTD was not statistically higher than PD (*χ^2^* = 0.086, *P* = 0.769).

**Table 3:** Multivariate Analysis of Risk Factors for PFS and OS.

	PFS			OS		
Risk Factor	HR	95%CI	*P*	HR	95%CI	*P*
Age	1.039	1.013–1.066	0.003	1.053	1.013–1.095	0.009
DS stage	1.735	1.166–2.582	0.007	1.914	1.012–3.621	0.046
ISS stage	0.992	0.685–1.437	0.967	0.726	0.387–1.364	0.320
FISH^a^	1.189	0.886–1.594	0.249	1.690	1.127–2.534	0.011
Regimens^b^	0.491	0.286–0.845	0.010	0.283	0.132–0.608	0.001
Cycles^c^ (N)	0.811	0.662–0.994	0.043	0.603	0.438–0.831	0.002

Abbreviations: OS, overall survival; PFS, progression-free survival; HR, hazard ratio; CI, confidence intervals;

DS, Durie-Salmon; ISS, International Staging System; FISH, interphase fluorescence in situ hybridization;

a Patients with abnormalities of 13q14, 1q21, 14q32 and 17p13 compared with no FISH abnormalities.

b Three-drug combinations compared with PD.

c Patients with 3 number of cycles or more compared with less than 3 cycles.

At the time of analysis, 33 (15.3%) patients had died, including 10 patients who had received PTD, and 14 patients in the PD groups, 7 patients in the PCD group and 2 in the PAD group died from treatment-related issues and MM. The median OS of the 215 patients was not reached at 64 months in either treatment arm and there were significant differences among groups (*P* = 0.005; Fig. 1B). The median OS for the PD arm was 44.0 months (95% CI: 39.2–48.8 months) while other arms were not reached, but the median OS for PTD, PCD and PAD was significantly longer than PD (*P* = 0.015, 0.013, 0.023). Respective 3-year OS was 86.3±5.3%, 75.1±11.0%, 75.5±8.1%, 65.3±8.8% with PCD, PAD, PTD and PD regimens. The 3-year OS rate of PCD was significantly higher than PD (*χ^2^* = 7.456, *P* = 0.006), but this was not statistically significant compared to PAD and PTD (*χ^2^* = 2.680, 1.421, *P* = 0.102, 0.233). The 3-year OS rate for PAD and PTD were not statistically higher than PD (*χ^2^* = 1.253, 1.324, *P* = 0.263, 0.250). The 3-year OS rate of PAD was not statistically higher than PTD (*χ^2^* = 0.042, *P* = 0.838). Comparing multivariate analysis of risk factors for PFS and OS, we found that patient’s age at diagnosis, a higher D-S stage and the number of induction therapy cycles significantly affected patients’ PFS and OS, certain cytogenetic abnormalities also affected OS, but these had little influence on PFS whereas ISS stage had no effect on PFS and OS (Table 3).

### Adverse Events

Treatment-related deaths in the subgroups included 15 patients (7.0% of total recruited patients), for whom infection-related deaths accounted for most (N = 12) of the case. No significant difference in treatment-related deaths was observed between groups. Fequently observed hematologic toxicities (Grade 3/4) were: thrombocytopenia (15.9%), neutropenia (14.0%) and anemia (7.9%). Among the patients in every subgroup, there was no significant difference observed in grade 3 to 4 hematological toxicities. The most common non-hematologic toxicities included (all Grades) peripheral neuropathy (55.5%), fatigue (26.8%), infection (23.2%), constipation (20.7%), herpes zoster (15.9%) and diarrhea (14.0%) (Table 4).

**Table 4:** Treatment-related adverse events.

	Total	PDT	PCD	PAD	PD
Adverse events, n(%)	(n = 215)	(n = 34)	(n = 77)	(n = 59)	(n = 45)
Hematologic events(3/4 grade)					
Neutropenia	23(14.0)	4(13.3)	11(16.7)	5(14.3)	3(9.1)
Thrombocytopenia	26(15.9)	6(20.0)	10(15.2)	6(17.1)	4(12.1)
Anemia	13(7.9)	3(10.0)	7(10.6)	2(5.7)	1(3.0)
Non-hematologic events (All grades)					
Fatigue	44(26.8)	10(33.3)	18(27.3)	9(25.7)	7(21.2)
Infection	38(23.2)	10(33.3)	15(22.7)	6(17.1)	7(21.2)
Constipation^a^	34(20.7)	13(43.3)	12(18.2)	5(14.3)	4(12.1)
Diarrhea^b^	23(14.0)	6(20.0)	12(18.2)	2(5.7)	3(9.1)
Pleural effusion and ascites	10(6.1)	4(13.3)	3(4.5)	1(2.9)	2(6.1)
Herpes zoster^c^	26(15.9)	12(40.0)	9(13.6)	3(8.6)	2(6.1)
Deep vein thrombosis	1(0.6)	1(3.3)	0(0.0)	0(0.0)	0(0.0)
Peripheral neuropathy^d^	91(55.5)	25(83.3)	34(51.5)	16(45.7)	16(48.5)
Grade 1	54(32.9)	11(36.7)	18(27.3)	13(37.1)	12(36.4)
Grade 2/3^e^	37(22.6)	14(46.7)	13(19.7)	5(14.3)	5(16.7)

a Incidence of constipation for the PTD arm was significantly higher than the PCD, PAD and PD groups (*χ^2^* = 5.002, 12.240, 9.876, *P* = 0.025, <0.001, 0.002).

b Incidence of diarrhea for the PTD arm was significantly higher than the PD group (*χ^2^* = 5.577, *P* = 0.048).

c Incidence of herpes zoster for the PTD arm was significantly higher than the PCD, PAD and PD groups (*χ^2^* = 8.568, 14.552, 12.641, *P* = 0.003, <0.001, <0.001).

d Peripheral neuropathy of all grades was more frequently reported in patients in the PTD group compared to the other groups which was obviously higher than that of the PCD, PAD and PD groups (*χ^2^* = 4.819, 18.848, 11.182, *P* = 0.028, <0.001, 0.001).

e Incidence of grade 2 to 3 peripheral neuropathy for the PTD arm was significantly higher than the PCD, PAD and PD groups (*χ^2^* = 7.562, 14.190, 9.584, *P* = 0.006, <0.001, 0.002). There was no significant difference in other treatment-related adverse events among groups.

The incidence of constipation for PTD arm was significantly higher than PCD, PAD, PD groups (*χ^2^* = 5.002, 12.240, 9.876, *P* = 0.025, <0.001, 0.002). The incidence of diarrhea for PTD arm was significantly higher than PD groups (*χ^2^* = 5.577, *P* = 0.048). The incidence of herpes zoster in the PTD was 40.0% which was significant higher than PCD (13.6%), PAD (8.6%) and PD (6.1%) group (*χ^2^* = 8.568, 14.552, 12.641, *P* = 0.003, <0.001, <0.001), respectively). Peripheral neuropathy of all grades was more frequently reported in patients in the PTD group compared to the PD, PCD and PAD groups (83.3% vs 51.5%, 45.7%, 48.5%) (*χ^2^* = 4.819, 18.848, 11.182, *P* = 0.028, <0.001, 0.001). The incidence of grade 2 to 3 peripheral neuropathy for PTD arm was significantly higher than PCD, PAD and PD groups (*χ^2^* = 7.562, 14.190, 9.584, *P* = 0.006, <0.001, 0.002). There was no significant difference in other treatment-related adverse events among groups.

## Discussion

MM is one of the most frequently observed hematologic cancers With an incidence in China of 1–2 per 100,000. Patients who can achieve CR are reported to be significantly improved with respect to PFS and OS whether in the patients received high-dose chemotherapy with ASCT or without ASCT, or relapse/refractory patients [7], [9]–[13]. Recently years, many prospective randomized clinical trials have pushed forth advances in the treatment of MM with targeted drugs, such as bortezomib-, thalidomide-, and lenalidomie-based regimens, effects of which have been reported to be significantly better than traditional therapies [1]–[6]. However, few clinical trials have compared regimens to develop a strategy to direct initial MM treatment, especially for the OS and patient quality of life [14].

At this time, regimens in China are mainly bortezomib-based therapies, including doublet regimens (PD), and triplet regimes such as PCD, PAD and PTD. Bortezomib, as a component of these protocols is given 1.3 mg/m^2^ twice a week, or 1.5 mg/m^2^ once a week. Dexamethasone is given at 160 mg for every course and sometimes as high as 480 mg every course. In our analysis, bortezomib was given twice a week and dexamethasone was administered at 160 mg for every course. ORRs reached 65–88% for the PD regimen, including at least 30–40% VGPR and about 20% CR/nCR [3], [15]–[17]. A phase 3 clinical trial IFM 2005-01 reported that in newly diagnosed patients who were suitable for ASCT, the ORR, CR/nCR, and effects better than VGPR for PD was 78.5%, 14.8% and 37.7%, respectively. The same results were observed in patients with poor disease stage or with adverse chromosomal abnormalities [3]. However, PD offered slight advantages with respect to PFS and OS but these were not statistically significant. The ORR of patients who received PD in our study was 77.8%, including 20.0% with CR/nCR and 17.8% of VGPR as reported.

Adding another drug to the PD regimen, such as an immunomodulatory agent, thalidomide or a conventional chemotherapeutic such as adriamycin or cyclophosphamide can achieve ORR as high as 90% with at least 60–70% VGPR and 40–50% CR/nCR [7], [18], [19]–[22]. In our study patients received treatment with median cycles of three drugs and above 30% cases only received 1 or 2 cycles, among which the PCD regimen had the optimal efficacy. With ORR, VGPR and CR/nCR rates for PCD of 97.4%, 27.3%, and 36.4%, these treatment modalities were all superior to PD. The effect of PAD was similar to PCD but PTD was only slightly better than PD. Because Cyclophosphamide has fewer adverse effects and is less expensive, a PCD regimen should gain favorable attention over time. In a phase II clinical trial [18] ORR, VGPR or better and CR/nCR rates were better for patients who received total four courses of PCD (28 days with every course, bortezomib was used twice per week, CTX at 1,200 mg/m^2^, dexamethasone at 480 mg every course). ORR, VGPR or better and CR/nCR rates were 88%, 61% and 39% respectively and the treatment onset of action was rapid. We observed similar effects with PCD. However, PCD with bortezomib once a week and less dexamethasone has similar effects with less adverse reaction [22].

Three-drug combinations were more effective than PD regimen, and for PFS, a three drug combination was better and OS was superior to PD. The median OS for the PD arm was 44.0 months while other arms were not reached, Respective 3-year OS was 86.3%, 75.1%, 75.5%, 65.3% with PCD, PAD, PTD and PD regimens, respectively. Because ours was only a retrospective study and the data can be affected by many factors although all the treatment center reach the consensus of MM. Therefore, further prospective randomized clinical trials are needed to confirm the induction treatment effect on PFS and OS.

At present, prognostic factors of patients with MM include host factors, such as age, abnormal cytogenetics, D-S stage and ISS stage [23]. ISS stage was derived from more than 11,000 patients and based on serum beta 2-microglobulin and albumin measurements and this criteria defines three risk groups with median survivals of 62, 44 and 29 months, respetcively [24]. In our study, ISS appears to be less helpful for predicting PFS and OS in Chinese populations [25], the results suggested that ISS had limitation when used for MM patients in China, but further randomized clinical trals are required to confirm this assertion.

Combination therapies based on bortezomib do not appear to cause serious adverse events [3]–[7], [15]–[22], and we found this to be true as well. The most common non-hematologic toxicities included peripheral neuropathy, fatigue, infection, constipation, herpes zoster and diarrhea. Herpes zoster was documented in 5–13% of cases in the US and Europe [6], [15], [26], but this was higher in Asia [26]–[31]. In our study, the incidence of herpes zoster was 41.4% in patients of the PTD group without routine anti-viral therapy, but much more lower in other groups with anti-viral therapy. No serious adverse events emerged, so short-term treatment with a low dose may be safe and effective [26], [32].

Peripheral neuropathy (PN) is another frequent adverse event of bortezomib but it is dose-limited and reversible [33]–[37]. However, how it occurs is not clear. The incidence of PN was higher in this study compared to reports from the US and Europe [15]–[19], [33]–[36], and this was true in the PTD group but whether bortezomib combined with thalidomide increased the PN incidence and its severity remained uncertain [20], [21], [33]–[36]. In our study, PN of a grade 2 or higher was observed in nearly half of the patients who received the PTD regimen, a finding that was significantly higher than in other groups. And the discontinuation rate of thalidomide maintenance in PTD group was higher than PCD, PAD and PD. In fact, this may be a potential contributor to the reduced PFS in PTD group versus other triplet agent therapies. further prospective studies expanding the number of cases should be performed for a more reliable result. However, there were no significant differences among PAD, PCD and PD groups.

Deep vein thrombosis (DVT) is another common treatment-related adverse event with bortezomib and other agents and it is observed in 3–7% of patients in the US and Europe [18], [38]. Patients who received PTD have a higher risk of DVT. In our study, routine anti-coagulation or anti-thrombosis agents were not used, and only one patient suffered from DVT/PE but did well with treatment. This frequency may be related to race and particulars of the local population and should be further studied to draw conclusions.

In conclusion, a three-drug combination is superior to bortezomib and dexamethasone, and PAD and PCD regimens are more efficacious with fewer adverse reactions, and the therapy is well-tolerated. In terms of the occurrence frequency and degree of PN, PAD and PCD are superior to PTD, especially the PCD. Considering drug toxicity, convenience and expense, we recommend a PCD scheme as a first-line therapy for MM for initial treatment.

## Supporting Information

Table S1 **Number of cycles and response rates.** Response for four regimens after every cycle are depicted in the Table.
(DOC)

Data S1 (PDF)

## References

[1] Kyle RA, Rajkumar SV (2008) Multiple myeloma. Blood. 111(6): 2962–2972. 10.1182/blood-2007-10-078022 PMC2265446 18332230

[2] Cavo ME, Zamagni P, Tosi P, Tacchetti C, Cellini D, et al (2005) “Superiority of thalidomide and dexamethasone over vincristine-doxorubicindexamethasone (VAD) as primary therapy in preparation for autologous transplantation for multiple myeloma.”. Blood 106(1): 35–39. 15761019 10.1182/blood-2005-02-0522

[3] Harousseau JL, Attal M, Avet-Loiseau H, Marit G, Caillot D, et al (2010) Bortezomib plus dexamethasone is superior to vincristine plus doxorubicin plus dexamethasone as induction treatment prior to autologous stem-cell transplantation in newly diagnosed multiple myeloma: results of the IFM 2005-01 phase III trial. J Clin Oncol 28: 4621–4629. 20823406 10.1200/JCO.2009.27.9158

[4] Sonneveld P, Schmidt-Wolf IG, van der Holt B, El Jarari L, Bertsch U, et al (2012) Bortezomib induction and maintenance treatment in patients with newly diagnosed multiple myeloma: results of the randomized phase III HOVON-65/GMMG-HD4 trial. J Clin Oncol 30: 2946–2955. 22802322 10.1200/JCO.2011.39.6820

[5] Wang L, Ran X, Wang B, Sheng Z, Liu L (2012) Novel agents-based regimens as induction treatment prior to autologous stem-cell transplantation in newly diagnosed multiple myeloma: a meta-analysis of randomized controlled trials. Hematol Oncol 30: 57–61. 21809367 10.1002/hon.1007

[6] Harousseau JL, Palumbo A, Richardson PG, Schlag R, Dimopoulos MA, et al (2010) Superior outcomes associated with complete response in newly diagnosed multiple myeloma patients treated with nonintensive therapy: analysis of the phase 3 VISTA study of bortezomib plus melphalan-prednisone versus melphalan-prednisone. Blood 116: 3743–3750. 20628153 10.1182/blood-2010-03-275800

[7] De la Rubia J, Roig M (2011) Bortezomib for previously untreated multiple myeloma. Expert Rev Hematol. 4: 381–398. 10.1586/ehm.11.38 21801129

[8] Durie BG, Harousseau JL, Miguel JS, Blade J, Barlogie B, et al (2006) International uniform response criteria for multiple myeloma. Leukemia 20: 1467–1473. 16855634 10.1038/sj.leu.2404284

[9] van de Velde HJ, Liu X, Chen G, Cakana A, Deraedt W, et al (2007) Complete response correlates with long-term survival and progression-free survival in high-dose therapy in multiple myeloma. Haematologica 92: 1399–1406. 18024376 10.3324/haematol.11534

[10] Niesvizky R, Richardson PG, Rajkumar SV, Coleman M, Rosinol L, et al (2008) The relationship between quality of response and clinical benefit for patients treated on the bortezomib arm of the international, randomized, phase 3 APEX trial in relapsed multiple myeloma. Br J Haematol 143: 46–53. 18673366 10.1111/j.1365-2141.2008.07303.x

[11] Harousseau JL, Attal M, Avet-Loiseau H (2009) The role of complete response in multiple myeloma. Blood. 114(15): 3139–3146. 10.1182/blood-2009-03-201053 19638622

[12] Lahuerta JJ, Mateos MV, Martinez-Lopez J, Rosinol L, Sureda A, et al (2008) Influence of pre- and post-transplantation responses on outcome of patients with multiple myeloma: sequential improvement of response and achievement of complete response are associated with longer survival. J Clin Oncol 26: 5775–5782. 19001321 10.1200/JCO.2008.17.9721

[13] Barlogie B, Anaissie E, Haessler J, van Rhee F, Pineda-Roman M, et al (2008) Complete remission sustained 3 years from treatment initiation is a powerful surrogate for extended survival in multiple myeloma. Cancer 113: 355–359. 18470907 10.1002/cncr.23546

[14] Rajkumar SV. (2012) Doublets, triplets, or quadruplets of novel agents in newly diagnosed myeloma? Hematology Am Soc Hematol Educ Program. 354–361. 10.1182/asheducation-2012.1.354 23233604

[15] Harousseau JL, Attal M, Leleu X, Troncy J, Pegourie B, et al (2006) Bortezomib plus dexamethasone as induction treatment prior to autologous stem cell transplantation in patients with newly diagnosed multiple myeloma: results of an IFM phase II study. Haematologica 91: 1498–1505. 17043025

[16] Rosinol L, Oriol A, Mateos MV, Sureda A, Garcia-Sanchez P, et al (2007) Phase II PETHEMA trial of alternating bortezomib and dexamethasone as induction regimen before autologous stem-cell transplantation in younger patients with multiple myeloma: efficacy and clinical implications of tumor response kinetics. J Clin Oncol 25: 4452–4458. 17785704 10.1200/JCO.2007.12.3323

[17] Jagannath S, Durie BG, Wolf JL, Camacho ES, Irwin D, et al (2009) Extended follow-up of a phase 2 trial of bortezomib alone and in combination with dexamethasone for the frontline treatment of multiple myeloma. Br J Haematol 146: 619–626. 19622094 10.1111/j.1365-2141.2009.07803.x

[18] Reeder CB, Reece DE, Kukreti V, Chen C, Trudel S, et al (2009) Cyclophosphamide, bortezomib and dexamethasone induction for newly diagnosed multiple myeloma: high response rates in a phase II clinical trial. Leukemia 23: 1337–1341. 19225538 10.1038/leu.2009.26 PMC2711213

[19] Palumbo A, Gay F, Falco P, Crippa C, Montefusco V, et al (2010) Bortezomib as induction before autologous transplantation, followed by lenalidomide as consolidation-maintenance in untreated multiple myeloma patients. J Clin Oncol 28: 800–807. 20048187 10.1200/JCO.2009.22.7561

[20] Cavo M, Tacchetti P, Patriarca F, Petrucci MT, Pantani L, et al (2010) Bortezomib with thalidomide plus dexamethasone compared with thalidomide plus dexamethasone as induction therapy before, and consolidation therapy after, double autologous stem-cell transplantation in newly diagnosed multiple myeloma: a randomised phase 3 study. Lancet 376: 2075–2085. 21146205 10.1016/S0140-6736(10)61424-9

[21] Mateos MV, Oriol A, Martinez-Lopez J, Gutierrez N, Teruel AI, et al (2010) Bortezomib, melphalan, and prednisone versus bortezomib, thalidomide, and prednisone as induction therapy followed by maintenance treatment with bortezomib and thalidomide versus bortezomib and prednisone in elderly patients with untreated multiple myeloma: a randomised trial. Lancet Oncol 11: 934–941. 20739218 10.1016/S1470-2045(10)70187-X

[22] Reeder CB, Reece DE, Kukreti V, Chen C, Trudel S, et al (2010) Once- versus twice-weekly bortezomib induction therapy with CyBorD in newly diagnosed multiple myeloma. Blood 115: 3416–3417. 20413666 10.1182/blood-2010-02-271676

[23] Bladé J, Rosiñol L, Cibeira MT (2008) Prognostic factors for multiple myeloma in the era of novel agents. Ann Oncol. 7: 117–120. 10.1093/annonc/mdn437 18790932

[24] Greipp PR, San Miguel J, Durie BG, Crowley JJ, Barlogie B, et al (2005) International staging system for multiple myeloma. J Clin Oncol 23: 3412–3420. 15809451 10.1200/JCO.2005.04.242

[25] Tao ZF, Fu WJ, Yuan ZG, Wang DX, Chen YB, et al (2007) Prognostic factors and staging systems of multiple myeloma. Chin Med J (Engl) 120: 1655–1658. 17935664

[26] Chanan-Khan A, Sonneveld P, Schuster MW, Stadtmauer EA, Facon T, et al (2008) Analysis of herpes zoster events among bortezomib-treated patients in the phase III APEX study. J Clin Oncol 26: 4784–4790. 18711175 10.1200/JCO.2007.14.9641

[27] Aoki T, Nishiyama T, Imahashi N, Kitamura K (2011) Efficacy of continuous, daily, oral, ultra-low-dose 200 mg acyclovir to prevent herpes zoster events among bortezomib-treated patients: a report from retrospective study. Jpn J Clin Oncol 41: 876–881. 21616919 10.1093/jjco/hyr063

[28] Tong Y, Qian J, Li Y, Meng H, Jin J (2007) The high incidence of varicella herpes zoster with the use of bortezomib in 10 patients. Am J Hematol 82: 403–404. 17133426 10.1002/ajh.20838

[29] Ohguchi H, Sugawara T, Ishikawa I, Okuda M, Tomiya Y, et al (2009) A retrospective analysis of bortezomib therapy for Japanese patients with relapsed or refractory multiple myeloma: beta2-microglobulin associated with time to progression. Int J Hematol 89: 342–347. 19296199 10.1007/s12185-009-0279-4

[30] Zheng W, Wei G, Ye X, He J, Li L, et al (2009) Bortezomib in combination with dexamethasone and subsequent thalidomide for newly-diagnosed multiple myeloma: a Chinese experience. Leuk Res 33: 1615–1618. 19773080 10.1016/j.leukres.2009.04.006

[31] Kim SJ, Kim K, Kim BS, Lee HJ, Kim H, et al (2008) Bortezomib and the increased incidence of herpes zoster in patients with multiple myeloma. Clin Lymphoma Myeloma 8: 237–240. 18765311 10.3816/CLM.2008.n.031

[32] Vickrey E, Allen S, Mehta J, Singhal S (2009) Acyclovir to prevent reactivation of varicella zoster virus (herpes zoster) in multiple myeloma patients receiving bortezomib therapy. Cancer 115: 229–232. 19090004 10.1002/cncr.24006

[33] Chaudhry V, Cornblath DR, Polydefkis M, Ferguson A, Borrello I (2008) Characteristics of bortezomib- and thalidomide-induced peripheral neuropathy. J Peripher Nerv Syst 13: 275–282. 19192067 10.1111/j.1529-8027.2008.00193.x PMC3741683

[34] Badros A, Goloubeva O, Dalal JS, Can I, Thompson J, et al (2007) Neurotoxicity of bortezomib therapy in multiple myeloma: a single-center experience and review of the literature. Cancer 110: 1042–1049. 17654660 10.1002/cncr.22921

[35] Chaudhry V, Cornblath DR, Polydefkis M, Ferguson A, Borrello I (2008) Characteristics of bortezomib- and thalidomide-induced peripheral neuropathy. J Peripher Nerv Syst 13: 275–282. 19192067 10.1111/j.1529-8027.2008.00193.x PMC3741683

[36] Richardson PG, Sonneveld P, Schuster MW, Stadtmauer EA, Facon T, et al (2009) Reversibility of symptomatic peripheral neuropathy with bortezomib in the phase III APEX trial in relapsed multiple myeloma: impact of a dose-modification guideline. Br J Haematol 144: 895–903. 19170677 10.1111/j.1365-2141.2008.07573.x

[37] Mohty B, El-Cheikh J, Yakoub-Agha I, Moreau P, Harousseau JL, et al (2010) Peripheral neuropathy and new treatments for multiple myeloma: background and practical recommendations. Haematologica 95: 311–319. 20139393 10.3324/haematol.2009.012674 PMC2817035

[38] Palumbo A, Gay F, Bringhen S, Falcone A, Pescosta N, et al (2008) Bortezomib, doxorubicin and dexamethasone in advanced multiple myeloma. Ann Oncol 19: 1160–1165. 18326520 10.1093/annonc/mdn018

