# Supplementary material for: The Choice of Regimens Based on Bortezomib for Patients with Newly Diagnosed Multiple Myeloma
Source: PLoS One. 2014 Jun 11;9(6):e99174. doi: 10.1371/journal.pone.0099174 (PMC4053437; doi:10.1371/journal.pone.0099174)
Supplement: Table S1 — Number of cycles and response rates. Response for four regimens after every cycle are depicted in the Table. (DOC) [file pone.0099174.s001.doc]

Supplemental Table

Number of cycles and response rates

|  | Total | PCD | PAD | PDT | PD |
| --- | --- | --- | --- | --- | --- |
| Cycles and response | (n=215) | （n=77） | （n=59） | （n=34） | （n=45） |
| Number of cycles  Median (range) | 3(1-8) | 3 (1-5) | 3(1-8) | 3(1-5) | 3(1-6) |
|  | 1^st^ cycle | | | | |
| Response n(%) | 215(100.0) | 77(100.0) | 59(100.0) | 34(100.0) | 45(100.0) |
| ORR | 166(77.2) | 66(85.7) | 50(84.7) | 21(61.8) | 29(64.4) |
| PR | 134(62.3) | 44(57.1) | 44(74.6) | 18(52.9) | 28(62.2) |
| VGPR | 27(12.6) | 18(23.4) | 5(8.5) | 3(8.8) | 1(2.2) |
| CR/nCR | 5(2.3) | 4(5.2) | 1(1.7) | 1(2.9) | 0(0.0) |
|  | 2^nd^ cycle | | | | |
| Response n(%) | 179(83.5) | 63(83.3) | 53(83.3) | 27(91.4) | 36(75.8) |
| OR | 161(74.9) | 61(79.2) | 49(83.1) | 21(61.8) | 30(66.7) |
| PR | 97(45.1) | 29(37.7) | 32(54.2) | 15(44.1) | 21(46.7) |
| VGPR | 52(24.2) | 24(31.2) | 15(25.4) | 6(17.6) | 7(15.6) |
| CR/nCR | 12(5.6) | 8(10.4) | 2(3.4) | 0(0.0) | 2(4.4) |
| 3^rd^ cycle | | | | | |
| Response n(%) | 138(63.4) | 47(63.3) | 48(63.6) | 16(82.9) | 27(60.6) |
| OR | 128(92.8) | 46(97.9) | 44(91.7) | 14(87.5) | 24(88.9) |
| PR | 48(34.8) | 16(34.0) | 14(29.2) | 6(37.5) | 12(44.4) |
| VGPR | 57(41.3) | 16(34.0) | 23(47.9) | 8(50.0) | 10(37.0) |
| CR/nCR | 23(16.7) | 14(29.8) | 7(14.6) | 0(0.0) | 2(7.4) |
| 4^th^ cycle or more | | | | | |
| Response n(%) | 83(34.1) | 23(27.6) | 33(30.3) | 9(42.9) | 18(36.4) |
| OR | 78(94.0) | 23(100.0) | 31(93.9) | 9(100.0) | 15(83.3) |
| PR | 17(20.5) | 6(26.1) | 5(15.2) | 1(11.1) | 5(27.8) |
| VGPR | 22(26.5) | 3(13.0) | 11(33.3) | 4(44.4) | 4(22.2) |
| CR/nCR | 39(47.0) | 14(60.9) | 15(45.5) | 4(44.4) | 6(33.3) |
