# Supplementary material for: The Choice of Regimens Based on Bortezomib for Patients with Newly Diagnosed Multiple Myeloma
Source: PLoS One. 2014 Jun 11;9(6):e99174. doi: 10.1371/journal.pone.0099174 (PMC4053437; doi:10.1371/journal.pone.0099174)
Supplement: Data S1 — (PDF) [file pone.0099174.s002.pdf]

	性别	年龄	类型	轻钅	M蛋白	ISS	DS	白蛋白	β2	原浆	幼浆	成浆	Hb	PLT	钙
1	F	59	A	K	5600	2	3A	37.3	5.099	1.5	6	7	68.5	223	2.1
2	M	57	A	K	8350	2	3A	28.5	3.698	48	45	0.5	79.9	74	2.4
3	M	53	G	L	9350	3	3B	24.3	15.803	60	14	4.5	53	166	2.3
4	M	44	G	K	1600	3	3B	30.4	13.863	2.5	46	9.5	74	81	2.15
5	M	48	G	L	1480	3	3A	36.3	5.607	0.5	22.5	13.5	100	163	2.03
6	F	67	G	K	4070	2	2A	36.7	4.68	11.5	5.5	12	89.1	170	2.33
7	M	55	G	K	7990	2	3A	29.7	3.897	0	2.5	4.5	121	247	2.02
8	F	53	G	K	5940	3	3B	41.2	6.51	7.5	15.5	9	77	315	2.19
9	M	39	G	L	8900	3	3B	27.5	5.8	10	30	10	95	158	2.95
10	F	54	G	L	15300	1	1A	43.5	2.012	19	13	3	115	163	2.28
11	M	63	A	K	6060	2	3A	33.5	4.227	6.5	23.5	11	92.4	155	2.2
12	M	50	G	K	8670	2	3A	31.9	3.058	13	23	4.5	86	116	2.1
13	F	55	G	K	7940	2	3A	35.4	5.13	21	41	9	110	39	2.18
14	F	54	G	K	3850	1	1A	37.9	2.312	26	20	4.5	130	62	2.14
15	M	45	A	K	6410	3	3A	33.2	5.796	35	0.5	1.5	92.3	95	2.16
16	M	72	G	K	7630	2	3A	29.1	2.431	6	22.5	4	117	160	2
17	F	76	A	L	4710	3	3A	26.5	7.259	20.5	3.5	0.5	55	50	2.37
18	M	73	L	K	2760	2	2A	32.4	3.209	3	12	5	106	16	1.88
19	M	59	G	K	9290	3	3B	26	80	2	20.5	10	90.4	54	2.16
20	M	75	G	K	6490	2	2A	36.1	4.458	23	19	1	138	132	1.6
21	M	67	G	L	8270	2	3A	29.1	4.3	27.5	20	2	71.7	197	1.98
22	M	59	G	L	11600	3	3A	28.2	7.438	20.5	25.5	2	75.7	114	2.64
23	M	65	G	L	5870	3	3B	24.7	6.037	10	20	10	76.4	186	1.93
24	M	78	L	L	6740	3	3B	31.3	42.875	2.5	6.5	1.5	89.1	274	2.29
25	M	63	G	L	4670	1	2A	42.5	2.181	0.5	2	4	140	163	2.47
26	M	51	G	K	7360	2	3A	34.1	2.331	0	6	2	102	56	2.06
27	M	59	A	K	6750	1	3A	36.5	2.42	0	4.5	7.5	91	183	2.19
28	M	66	G	L	9700	2	3A	32.9	5.237	13	27	4	81	178	2.12
29	M	55	L	L		3	3A	31	6.98	12	3.5	0.5	102	160	2
30	女	51	L	L	1530	3	3B	31.8	22.15	20	14.5	0	60	169	2.25
31	M	50	D	L		3	3B	41.3	5.642	32	14	9.5	82	136	2.98
32	F	61	G	K	9700	2	3B	29.9	3.197	12	1	2	69	112	1.98
33	M	52	A	K	3130	2	3A	43.4	4.53	4	3	5	74.8	40	2.2
34	F	66	G	L	4120	2	2A	41.7	3.6	7	2	4	64.7	162	2.23
35	F	57	G	K	3860	2	3A	26.1	4.019	3.5	12	22.5	58	129	2.57
36	F	64	G	K	19400	1	2A	38.5	1.868	3.5	10	13	104	229	2.47
37	M	50	D	L		1	2A	41.4	2.638	23	56	7	51.3	74	2.12
38	F	56	G	K	3700	1	1A	39.3	1.78	9	22	5.5	112	250	2.33
39	F	79	L	L	5320	1	2A	43.2	1.922	27	19	1	84.8	268	2.31
40	M	61	G	L	5590	2	3A	30.2	3.053	8	39	10	44	27	2.01
41	F	64	G	K	8980	2	3A	27.6	4.41	0	2	0	67	108	2.03
42	F	64	L	L		3	3A	38.7	7.35	12	19	25.5	73	52	2.06
43	M	80	G	L	3740	3	3B	22.9	11.49	2	11	4	78	98	1.83
44	M	57	G	K	4100	3	3B	38.1	15.3	2	14	5	63.5	80	2.08
45	M	67	G	L	1360	3	3B	37.2	49.037	64	9.5	0	76.3	99	2.26
46	F	70	A	K	4350	1	2A	41	1.92	8	15	5	99	285	2.63
47	M	81	A	L	4000	1	2A	40.5	2.555	3.5	4.5	7	110	142	2.16
48	M	31	G	L	5820	3	3A	27.1	9.87	13	7	12	71	198	2.21
49	M	73	L	K	269	2	2A	30.8	2.349	10	4	4	77.3	104	2.05
50	M	70	L	L	2430	2	3A	42.7	3.89	13	3	5	87.6	147	2.11
51	M	70	A	K	2120	3	3B	27.6	11.528	14	2	2			2.01
52	M	49	L	L	1430	3	3B	37.2	13.35	23	12.5	11.5	69	142	2.62
53	M	78	G	K	1150	3	3B	35.1	8.301	8	15	7	63	257	2.06
54	M	59	D	L		3	3B	41.5		54	6	6	53.9	120	2.41
55	F	57	A	L		1	3A	49.3		55	20	6	89	163	2.31
56	M	57	L	L	812	3	3B	37	9.705	9	17	1	50.2	51	1.97

57	M	53	A	K	4000	2	3A	35.8	4.418	4	14	2	100.5	233	2.15
58	M	53	G	K	9350	3	3B	24.3	15.803	30	31	1	53	166	
59	F	56	A	L	4390	3	3A	24.4	4.925	28	30	2	111	146	2.47
60	M	59	G	K	4680	2	3B	33	5.06	5	4.5	5.5	95	245	2.8
61	M	52	L	K		2	2A	42.8	3.93	26	1	6	121	321	2.2
62	M	73	A	K	7390	1	3A			14	3	1	65	230	2.31
63	M	70	A	K	7210	3	3A	20	1.962	20	14	7	69	143	2.2
64	M	73	A	L	4630	1	2A	37.1	1.997	23	4	3	96	80	2.21
65	M	65	A	K	2150	2	3A	34	3.563	2	3	9	76	181	2.09
66	M	76	G	K	9400	3	3A	17.9	12.45	0.5	1.5	4.5	84	61	2.51
67	M	80	G	L	15751	3	3A	47.1	10	3	2	2	90	215	1.8
68	F	83	A		4790	2	2A	34.8	2.57	4	4	2	80	200	2
69	F	57	L	L	520	1	2A	31	2.453	11	15	13	112	200	2.23
70	M	49	N			3	3A	47.6	6.93	23	10	3	138.4	166	2.19
71	F	66	G		5620	1	2A	37	5.748	17	5	4.5	93.4	156	2.2
72	F	62	L	L	1130	2	3A	26.4	2.1	10	4	1	69.6	281	2.9
73	M	80	G	L	9150	3	3B	29.3	12.611	7	8	3	92.3	122	1.78
74	M	56	G	L	12600	3	3A	35	12.607	4	9	4	75	140	2
75	F	75	A	L	1220	1	3B	36.5	1.815	24	2	1	75	177	2.39
76	M	61	G	L	2190	1	1A	39.5	2.721	7	8	8	148	124	2.18
77	M	45	L	K	1320	2	1A	49.4	4.98	5	3	8	129	172	2.26
78	M	46	G	K	7500	3	3A	23	7.1	34	24	1	75	142	2.1
79	M	65	G		5190	1	3A	35.4	1.9	23	2	2.5	124.4	146	1.97
80	F	56	G	L	4070	2	1A	29.6	1.427	9	8	7	103	331	2.24
81	F	59	A	K	5450	2	3A	41.3	3.636	9.5	35	5	66	207	2.27
82	F	58	G	L	11200	2	3A	29.9	3.912	1.5	44.5	8	86	189	2.02
83	M	48	L	L	1270	1	2A	51.1	3.092	17.5	50	1	144	160	2.12
84	M	67	A	K	5590	1	3A	35.2	2.567	45	26	7	61	113	2.09
85	M	58	G	K	2560	1	3A	46.8	1.659	1	1.5	7.5	150	149	2.22
86	F	59	L	L	753	2	3A	49.2	4.74	0	8.5	4.5	107	194	2.26
87	M	74	L	L	780	2	3A	42.5	3.78	2	35	4.5	97	188	1.97
88	M	73	A	K	4300	2	3A	23.1	2.321	16	12	1.5	87	99	2.48
89	M	53	L	K	714	1	2A	46.4	2.49	0	1	2	129	99	2.19
90	M	37	A	L	7610	2	3A	30.9	3.514	0	0	3	58.1	181	2.13
91	F	38	G	L	3250	1	3A	38.7	2.138	22	30	2.5	62	278	2.17
92	F	53	G	L	1870	3	3A	34.8	6.093	9.5	38.5	3	59	166	2.26
93	M	56	L	K	104	3	3B	42	7.65	1	21.5	3	91	74	3.47
94	F	64	A	L	6620	2	3A	32.6	4.376	16	13	1	55.2	282	2.01
95	M	54	G	K	1740	1	3A	36	2.238	2	0	1	91	243	2.01
96	F	61	G	K	6390	3	3B	35.1	13.753	7.5	3.5	23	61	131	2.64
97	M	66	L	K	1000	1	3A	46.5	2.914	13.5	6.5	24	132	132	2.34
98	M	66	G	K	5750	2	2A	34.7	3.76	10.5	13.5	6.5	111	91	2.12
99	F	56	G	L	3110	2	3A	48.2	3.808	0.5	1	2	121	162	2.22
##	M	55	G	K	10100	3	3A	24.4	9.799	25.5	15	9.5	49	143	1.82
##	M	51	G	L	11000	3	3A	28.2	8.67	4.5	8	33.5	59	157	2.4
##	M	51	A	K	6020	3	3B	31.6	18.194	7.5	9	12.5	70	139	3.27
##	M	51	G	L	4830	2	3A	34.2	3.542	13.5	8.5	3	69	163	1.8
##	M	60	A	K	1261	1	1A	40.8	1.78	0	0	1.5	121	219	2.01
##	M	78	L	K	1400	3	3B	30	11.52	35.5	12	3	99	55	2.72
##	F	76	A	K	5111	3	3A	26.1	1.452	18	36	3.5	71	166	2.96
##	F	56	L	K	8250	1	3A	35.8	1.71	30.5	20	12.5	113	184	2.01
##	M	66	L	L	2020	2	2A	45.3	5.43	10	25	11	130	253	2.52
##	M	63	A	L	4570	2	2A	32.4	3.63	1.5	2	5.5	99	173	2.15
##	M	60	G	L	3870	1	3A	35.1	1.98	1	1	5	64	121	2.31
##	M	63	G	K	3320	2	3A	40.4	5.46	20	26	2	97	269	1.94
##	M	75	A	L	2010	3	3B	42.9	10.98	5	20	4	91	145	2.33

##	F	57	A	L	2490	2	2A	19.1	2.692	6			86.4	80	1.96
##	M	61	G	K	2660	1	2A	50.5	1.576	6.5	5.5	3	145	179	2.22
##	F	69	G	K	6540	2	2A	32.7	3.523	0.5	6.5	18.5	115.1	211	2.35
##	M	66	L	K	1000	1	3A	41.8	3.49	2	25	54	96.3	165	2.27
##	F	72	A	L	1950	2	3A	32.2	4.203	40.5	17	5.5	62	99	2.01
##	F	63	L	L	1990	1	3A	50.4	2.038	3.5	30	11	89.9	187	2.61
##	F	66	G	K	7120	3	3A	44	12.28	57	22	1	97	233	2.32
##	F	60	G	L	6760	2	3A	24.8	1.886	31	20	3.5	95.6	252	1.85
##	F	45	G	K	11400	3	3A	24	3.993	12.5	4	5.5	47	191	2
##	M	43	G	K	11700	3	3A	25.4	11.85	18	20	0	53.1	33	2
##	M	59	N			3	3B	51.8	9.369	74			101	79	3
##	M	67	L	L	1090	3	3B	46.2	9.682	3	8.5	3.5	89	158	2.16
##	M	53	A	L	1430	1	3A	43.1	9.231	8	15	0	85	106	2.26
##	M	60	A	K	5180	2	3A	34.7	4.585	11	26	1	104.4	168	2.2
##	F	63	A	K	4250	1	3A	37.9	2.135	10	30	4	109.7	196	2.14
##	M	70	A	L	4050	3	3B	176	7.005	13.5	28	3	104.6	207	3.37
##	M	71	A	K	1790	1	3A	43	2.28	6	14.5	4.5	127.8	191	1.98
##	F	55	G	K	6360	1	2A	45.6	2.767	24	50	0	88.3	209	2.28
##	M	63	L	K	1140	1	3A	36.2	1.958	2	11.5	0	116	311	2.02
##	F	48	L	L	1250	3	3A	42.7	10.428	39	28	1.5	93.4	181	2.14
##	M	64	A	K	2970	1	2A	42.1	2.548	3	9	1	102	164	2.16
##	F	49	A	L	4490	3	3A	44	7.641	14	10	1	76.7	322	2.42
##	F	54	G	K	7780	3	3A	29.7	12.459	5	5.5		72	93	2.2
##	F	38	G	K	6900	3	3A	21.9	6.78	35	37.5	4	67	259	1.86
##	F	81	L	K	3130	3	3A	40	3.594	15	24	10	81	115	2.29
##	M	80	G	K	9150	2	3A	28.1	3.88	0	2	13	103	39	1.88
##	M	66	L	L	1730	3	3B	44.4	28.5	15	10.5	32	119	145	3.08
##	F	59	G	L	5160	3	3B	30.2	56.21	0	0	6	37	24	1.95
##	M	67	A	L	5720	3	3A	44.3	1.63	2	10	26	53	110	2.27
##	F	49	G	K	6890	2	3A	32.9	3.11	10	12.5	0	57	76	2.09
##	M	53	G	L	7020	2	3B	29	2.98	7	15	10	60	112	1.98
##	M	76	A	L	4630	3	3B	43.3	5.6	2	9	21	75	80	2.09
##	M	69	G	K	17400	2	3A	34.7	1.8	3	7	1	118.4	143	1.87
##	F	56	A	L	2430	1	3A	38.6	1.8	4	12	4	60.7	124	2.26
##	M	60	L	K	5150	2	3B	49.3	4.8	15	13.5	17.5	73.8	105	2.22
##	F	62	L	L	935	3	3B	34.9	18.327	13	3	12	95	146	2.52
##	M	61	G	L	8050	3	3B	21	15.321	12	7	9	88	160	2.06
##	F	56	L	L	6090	3	3A	41.8	7.428	16	8.5	2	102.2	184	2.99
##	M	57	G	L	9040	2	3A	34.8	4.588	10	9	5	76.3	159	2.22
##	M	75	A	K	3830	2	3B	33.9	3.616	6.5	10	1	98.7	139	3.63
##	F	64	L	L	469	3	3B	50.5	8	7	5	3	53.5	130	2.01
##	F	50	L	L	1160	3	3B	50.7	15.1357	12	3	5	75.9	152	3.32
##	M	73	G	L	2980	2	3A	33.3	2.8	11	6	1	123	242	2.13
##	M	64	G	K	12000	2	3A	31.7	1.2	10	2	3	59	99	2.2
##	F	53	A	L	6880	2	3A	30.8	2.756	9.5	8.5	10	90.5	99	2.25
##	M	60	A	L	5850	3	3A	34.1	10.008	2	1.5	1.5	79.1	131	2.28
##	M	53	G	K	2610	1	1A	40.8	1.665	4	4.5	2	126	112	2.02
##	M	63	L	k		2	3A	34.5	4.875	15	6	8.5	68.3	134	2.43
##	M	57	L	L	1030	3	3B	26.7	12.15	5	4.5	1.5	78.3	280	2.14
##	M	60	A	L	5590	2	3A	29.7	3.15	7	3	6	110	187	2.28
##	F	63	A	L	3170	3	3A	38.5	8.46	22.5	7.5	9.5	64	61	2.31
##	F	73	L	K	924	1	2A	52.3	1.383	7.5	10	25	109	158	2.81
##	F	61	G	L	3410	1	2A	40.8	2.46	12.5	9.5	1	135	253	2.28
##	M	62	G	L	5710	3	3B	39.8	10.073	10.5	3	1.5	100	109	3.14
##	F	46	L	L	2160	3	3B	38	11.493	13.5	10	7	67	142	2.62
##	F	64	D	L	1400	1	2A	48.8	4.063	24	16	8	81	99	2.08

##	F	56	L	K	527	2	2A	37.1	3.598	12	11	5	95	161	2.33
##	M	66	A	K	3110	2	2A	32.7	2.19	8	12	9	113	257	2.57
##	F	75	L	K	8000	3	3B	41.2	10.59	8.5	7.5	6	70	138	3.17
##	M	60	A	L	2830	3	3B	43.8	11.25	23	10	12	71	238	2.26
##	M	64	L	K		1	3A	45.1	1.239	24	33	17	116	239	2.36
##	M	61	G	K	8340	3	3A	25.2	9.03	50	7	5	74	230	2.12
##	F	62	L	L	5940	3	3A	36.4	5.66	23	31.5	1.5	100	92	2.29
##	F	65	G	K	6090	3	3A	36.9	15	54	15.5	0	80	173	2.07
##	M	48	G	K	6440	1	1A	35.5	1.56	13	6	1	117	294	2.12
##	M	64	G、A	L	5710	3	3B	20.5	9.93	40	6	2	68	113	2.21
##	F	59	A	L	7200	3	3A	28	7.95	10	9	38	76	44	1.9
##	M	55	A	K	5330	2	3A	29.7	2.73	6	2	79	79.4	244	2.25
##	F	67	G	L	10400	2	2A	30.8	2.667	3	10	2	73.4	179	1.64
##	M	74	A	K	1680	2	3A	28.9	2.366	26.5	20	3	83	193	2.39
##	F	68	G	K	13100	3	3A	26	5.766	2	16	0	70	101	1.98
##	M	64	G	K	4570	1	3A	37.1	5.5	29	31	8.5	103.3	129	2
##	M	49	G	L	4370	2	3A	30	5.076	6	15	3	104	75	
##	M	62	G	K	2180	3	3B	32.1	8.61	26	2	1	61	71	2.36
##	M	62	L	L	1060	3	3B	42.2	37.614	50	22	8	72	152	2.37
##	M	44	L	L	2940	3	3B	52.1	10.273	25	10	10	104	84	2.37
##	F	56	G	L	7190	2	3A	31.4	4.9	2	3.5	1	76	172	2.13
##	F	47	G	L	6370	2	2A	31.9	2.67	23	1	2	103	225	2.43
##	M	74	A	L	2170	1	2A	44.1	1.061	15	1	5	94	145	2.26
##	F	63	G	K	3800	1	3A	44.1	3.33	10	4	1	100	198	2.19
##	M	63	N			2	3A	42.8	9.9	7	3	3	65.9	64	2.23
##	M	55	L	L	6630	2	3A	47.6	4.917	6	12	2	114.4	93	2
##	M	44	A		6980	2	3B	33.8	2.585	5.5	5	1	62	174	1.9
##	M	60	G	L	7560	2	3A	33.6	2.634	8	9	2	73	427	2.09
##	M	55	G		19700	3	3A	29.8	6.686	9	12	9	58.3	106	1.92
##	F	50	G	K	3300	2	3A	43.4	3.69	3	14	1	95	130	2.27
##	F	58	G	K	1350	1	3A	46.4	2.772	4	12	5	96.3	283	2.47
##	M	59	G	L	12600	2	3A	21.3	2.136	7.5	3	5	68.9	68	2.31
##	M	59	L	L	1860	2	3A	31.8	1.6	2	10	8	78.4	116	2.32
##	M	67	A	L	3600	3	3B	37.5	19.238	5	4	1	58.4	189	2.34
##	M	59	A	K	1600	3	3B	37.4	8.43	9	8	2	80	183	2.15
##	M	60	G	L	2890	1	1A	37	3.35	12	2	1	134.8	220	2.2
##	F	58	G	L	2950	3	3A	34.8	8	3	8	4.5	79.3	176	2.12
##	M	60	L	L	30.4	1	3A	49.6	2.061	25	6	7	146	159	2.4
##	M	46	G	K	18800	2	3A	22	1.1	13	3	2	60.1	126	2.07
##	F	53	A	K	6480	3	3A	41.8	7.98	4	13	20	74	222	2.12
##	F	50	G	K	5000	2	2A	30.7	1.821	16	14	3	88	217	2.14
##	F	62	A	L	5530	2	3A	37	4.198	17	3	1	106.7	179	2.16
##	M	1	A	K	7490	3	3A	26.8	10.44	5	7	8	90	166	2.54
##	M	61	G	K	2690	3	3B	34.6	8.76	8	4	2	73	246	2.32
##	F	61	G	K	18300	2	3A	32.4	5.229	9	13	6	116	217	2.37
##	F	61	G	L	8700	2	3A	23.3	1.1	13	7	3	87	207	1.84
##	M	50	A		3340	1	3A	42.5	2.649	17	12	3	145	157	2.31

CRP	LDH	1	2	3	4	5	6	7	8	PFS	进展	OS	死亡
0.89	103	MR	PR	PR	VGPR					17	Y	20	Y
3	125	MR	PR	VGPR	CR					24	Y	64	N
7	295	MR	PR	VGPR	CR					21	Y	64	N
3.62	181	SD	SD	SD						5	Y	44	N
4.02	211	PR	VGPR	VGPR						50	Y	63	N
8.68	211	PR	VGPR							18	Y	57	N
10.78	144	PR	PR	PR						8	Y	63	N
4.37	221	PR	VGPR	VGPR						27	Y	60	N
6.65	169	MR	PR	PR	VGPR	VGPR				24	Y	44	N
0.07	136	MR	PR	VGPR	VGPR					46	N	46	N
4.01	113	PR	VGPR	VGPR	VGPR					32	Y	44	N
2.22	251	PR	PR	PR						9	Y	12	N
9	138	PR	PR							5	Y	13	Y
2.23	118	PR	PR							4	Y	40	N
0.22	119	MR	PR							24	Y	60	N
5.38	206	PR	PR							16	Y	35	Y
21.72	145	PR								15	Y	22	Y
4.01	689	PR	VGPR							6	Y	31	Y
10.94	348	PR								13	Y	59	N
3.18	92	PR	PR							12	Y	19	Y
231	7.92	SD	MR							3	Y	12	Y
0.39	122	VGPR								32	Y	42	N
1.07	117	PR								12	Y	31	Y
2.45	222	MR	MR							7	Y	12	Y
4.25	175	VGPR								16	Y	34	N
4.77	124	MR	PR							36	N	50	N
4.56	68	PR	VGPR	VGPR	CR					48	Y	51	N
1.91	129	MR	MR							6	Y	34	Y
3	145	PR	PR	VGPR	nCR					23	N	23	N
13.18	147	VGPR								9	N	9	N
1.3	225	SD	SD	SD						5	Y	40	N
0.8	101	PR	PR	PR						6	Y	15	N
3.9	569	SD	SD	PR	PR	PR				6	N	6	N
0.9	203	PR								2	N	2	N
9.65	318	SD	SD							2	Y	33	N
0.1	228	VGPR	VGPR	VGPR	CR					22	Y	42	Y
0.28	104	MR	PR	PR						25	N	25	N
0.56	146	SD	SD	SD	SD					14	Y	23	Y
1.07	266	PR	PR	PR	PR					44	N	44	Y
1.3	162	MR	PR	PR	VGPR					12	Y	29	N
1.24	320	PR	PR	VGPR	VGPR					8	Y	16	Y
2.25	198	SD	PR	VGPR	CR					27	N	27	N
203.76	112	SD								3	Y	12	Y
34.86	239	MR								3	N	3	Y
17.49	629	SD	MR							2	N	2	Y
3.67	133	PR	VGPR	VGPR	CR					8	Y	30	N
45	194	PR	VGPR							14	N	14	Y
25.7	156	PR	VGPR	CR						34	N	34	N
1.2	150	PR	VGPR	VGPR						12	Y	24	N
1.3	134	MR								4	Y	24	Y
3.2		PR								6	Y	24	Y
16.2		MR	PR	PR						3	N	3	Y
48.7	220	MR	PR	PR	PR					10	Y	14	Y
1.8	161	PR								2	N	2	Y
23	210	PR	PR	VGPR	CR					15	N	15	N
0.5	257	PR	PR							4	N	4	N

26.4	79	PR	VGPR	VGPR				3	N	3	N
13		PR	PR	VGPR	CR			40	Y	42	N
13.6	316	SD	SD	SD	PR	PR	PR	20	N	20	N
10	410	PR	PR	PR				30	Y	57	Y
4	154	PR	nCR					12	N	12	N
6	200	SD	SD	SD	SD	SD		5	Y	45	N
7	111	PR	PR	PR				24	Y	58	N
1	135	PR	PR					2	N	2	N
1.7	117.2	PD						6	N	6	N
6.09	147	PD						6	N	6	N
0.7	381	PR	PR	PR	PD			10	Y	11	N
1	140	PR	PR	VGPR	nCR			23	Y	28	N
11	139	PR	PR	PR	PR	PR	PR	26	N	26	N
1	366	PR	PR					2	N	2	N
1	269	PR	PR	PR	PR			44	Y	51	N
60.1	152	PR	VGPR					3	N	3	N
54.4	152	PR	PR	PR				6	Y	8	N
1	144	PR	nCR	nCR				8	N	8	N
1.1	202	PR						7	N	7	N
1	225	SD	SD					11	N	11	N
12	208	PR	PR	VGPR	VGPR			9	N	9	N
2	203	PR	PR	PR	VGPR			6	Y	33	N
0.3	135	PR						2	N	2	N
16.1	255	PR	PR	PR	CR			30.5	N	30.5	N
29.75	313	PR	PR	VGPR	CR			12	Y	23	Y
4.21	119	MR	PR	PR	PR			6	Y	28	N
2.69	170	SD	PR	PR	VGPR			26	N	26	N
5.53	103	PR	VGPR	VGPR				9	Y	17.5	Y
1.59	141	PR	VGPR	CR				32	N	32	N
5.26	152	PR	VGPR	VGPR	CR			23	Y	40	N
2.6	289	PR	VGPR	CR				20	Y	24	N
13.3	227	VGPR						20	Y	22.5	Y
1.49	218	CR	CR					35	N	35	N
0.48	152	PR	PR	VGPR				11	Y	29	Y
2.4	257	PR	VGPR					31	N	31	N
2.9	255	PR						5	Y	30.5	N
18.7	372	PR	PR	VGPR				10	Y	27	N
1.6	180	PR	CR	CR	CR			27	N	27	N
1.6	180	PR						6	N	12	N
2.6	129	PR	PR	PR				6	Y	28	N
8.2	204	MR	PR	VGPR	CR			8	Y	25.5	N
1.3	121	PR						5	Y	26	N
4.8	303	PR	VGPR	CR				20	N	20	N
0.3	125	SD						22.5	n	22.5	N
6.6	176	PR						9	N	9	N
3.9	75	MR	PR	PR				5	Y	20	N
1.3	252	PR	VGPR	VGPR				24	N	24	N
1.3	112	PR	VGPR					41	n	41	N
15.8	183	PR	PR	VGPR	VGPR			10	Y	28	Y
3.55	114	PR	VGPR	VGPR				13	Y	15	N
2.1	172	VGPR	VGPR	VGPR				6	N	6	N
27.4	220	VGPR	CR	CR	CR	CR		11	N	11	N
1.8	119	VGPR	nCR	nCR	CR			17	N	17	N
3.8	163	VGPR	VGPR	CR				18	N	18	N
7	243	PR						10	Y	13	Y
10	103	PR	PR	VGPR	CR			15.5	N	15.5	N

1	178	PR	VGPR			2	N	2	N
0.4	227	PR	PR	VGPR	CR	19	N	19	N
22.3	139	PR	PR			4.5	N	4.5	N
33.8	158	VGPR	nCR	nCR		12	N	12	N
10.2	178	SD	PR	PR	CR	12	Y	26	N
14	201	VGPR	VGPR	VGPR		13	N	13	N
23	152	PR	PR	PR	PR	17.5	N	17.5	N
1.2	141	PR	PR	VGPR		22	N	22	N
1	131	SD	PR	PR	PR	27.5	N	27.5	N
0.3	289	PR	PR	PR		10.5	N	10.5	N
14.9	200	PR	PR	CR	CR	7	Y	11	Y
5	144	SD	PR	PR	PR	16	N	16	N
1.4	212	VGPR	VGPR	CR		13	Y	27	N
20	103	VGPR	VGPR	CR		10.5	N	10.5	N
1	132	PR	VGPR			9.5	N	9.5	N
4	93	VGPR	VGPR			7.5	N	7.5	N
1.1	191	SD	VGPR	VGPR	CR	6	Y	9	N
0.8	119	PR	PR	PR	PR	14	N	14	N
1	123	VGPR	VGPR	CR		13	N	13	N
3.9	323	SD	SD	PR	VGPR	25	N	25	N
3.3	97	PR	PR			10.5	N	10.5	N
3.8	177	SD	SD	SD		2.5	N	2.5	N
3	200	PR	PR	PR		3	N	3	N
8.99	183	PR	PR	PR		5	N	5	N
1	130	PR	PR	PR	PR	11	Y	15	Y
1.64	139	CR	CR			9	N	9	N
1.71	188	PR				10	N	10	N
125.32	569	VGPR				13	N	13	N
2.11	183	CR				38	N	38	N
27	223	CR				7	Y	10	Y
8	432	VGPR	CR			12	Y	22	Y
63.5	159	PR	PR			2	N	2	N
26.8	186	PR				1	N	1	N
0.1	132	PR	VGPR			3	N	3	N
0.5	198	VGPR	VGPR			3	N	3	N
0.1	153	VGPR	VGPR	VGPR	CR	5	N	5	N
3.3	155	PR	PR	PR		7	N	7	N
13	160	VGPR	VGPR	CR	CR	11	N	11	N
1	233	PR	PR			12	N	12	N
	160	VGPR	VGPR			2	N	2	N
1.7	539	PR	PR			2	N	2	N
19.8	211	VGPR	CR	CR		5	N	5	N
1.5	262	PR				1	N	1	N
18.2	227	PR				3	N	3	N
1.8	146	PR	PR	VGPR	VGPR	14	Y	40	N
2.84	76	VGPR	VGPR	CR		24	Y	42	N
1.2	117	SD	SD	SD	SD	12	Y	37	N
0.2	219	VGPR	nCR	nCR		27	N	27	N
15.6	208	PR				5	Y	16	Y
1.6	122	PR	PR	PR	PR	6	Y	27	N
1.3	131	PR	VGPR	CR		27	N	26	N
1.1	191	PR	VGPR	CR	CR	8	Y	26	N
7.4	258	MR	PR	PR		7	Y	21	N
87.4	155	PR	PR	PR		4	Y	9	N
16.2	186	MR	PR	VGPR		12	Y	38	N
1.1	130	MR	PR	VGPR	CR	25	N	25	Y

3.08	218	MR	PR	PR	VGPR					23	N	23	N
147	198	PR	VGPR	CR						7	Y	30	N
9.1	230	VGPR	VGPR							10	Y	13	N
10.3	168	MR	PR	VGPR						11	N	11	N
1.9	217	CR	CR	CR						3	N	3	N
	129	PR	PR	PR	VGPR					16	Y	19	N
5	565	PR	PR	VGPR	VGPR	VGPR				15	N	15	N
	155	PR	PR	PR						15	N	15	N
	126	PR	PR	PR	PR	PR	PR	VGPR	CR	22	N	22	N
1	65	PR								10	N	10	N
20.5	144	PR								7	N	7	N
5	106	PR	VGPR	VGPR						5	N	5	N
	154	PR	PR	VGPR	VGPR					8	N	8	N
0.3	145	PR	VGPR	VGPR						15.5	Y	21	N
4.6	229	PR	PR							9	N	9	N
1	174	SD	SD	SD	SD					6	N	6	N
		PR	PR	VGPR	VGPR	VGPR				21	N	21	N
11.5	191	SD	SD	SD						14	N	14	N
	241	PR	VGPR	VGPR	VGPR	VGPR	CR			36	Y	36	Y
		PR	PR	PR	PR	VGPR	VGPR			29	N	29	N
0.8	169	PR	PR	VGPR	CR					23	N	23	N
	104	PR	PR							12	N	12	N
1.17	178.2	PR								6	N	6	N
1.1	164	PR	PR	PR	VGPR					13	N	13	N
0	217	PR	PR	PR	PR	PR	nCR			9	N	9	N
1	142	PR	PR	VGPR	CR					11	N	11	N
10.5	79	PR	PR	VGPR	CR					8	N	8	N
0.3	169	PR	PR	PR	PR					29	Y	36	N
0.8	116	PR	PR	VGPR	nCR					6	N	6	N
0.1	137	PR	PR	VGPR	nCR					9	N	9	N
0.2	156	PR								1	N	1	N
0.3	119	VGPR	VGPR	VGPR	CR					7	Y	9	N
0	175	VGPR	VGPR	VGPR						3	N	3	N
3	307	PR	VGPR	VGPR	CR					19	N	19	N
1	137	PR	PR	PR	PR					27	N	27	N
5.2	173	PR	PR	PR	PR					7	N	7	N
0.1	180	PR	PR	PR	PR	PR				5	N	5	N
	171	PR	VGPR	VGPR	VGPR	VGPR				10	N	10	N
27.6	100	PR	VGPR	VGPR	VGPR					4	N	4	N
	126	PR	PR	VGPR	VGPR					18	N	18	N
1	109	PR	PR							2	N	2	N
0.8	161	PR	VGPR	nCR	nCR					8	N	8	N
2	69	PR								2	N	2	N
1	243	SD	SD	SD						11	Y	23	N
1.1	245	PR	VGPR	VGPR	nCR					3	N	3	N
44.3	209	PR	PR							2	N	2	N
	213	PR	PR	VGPR	CR					11	N	11	N

初治日期	P53	1q21	IGH	D13S319	13q14RB1缺失
2007.11.7-2011.4.28					
2007.4.3-2012.5.31	1	1			
2007.3.30-2009.1.10					
2007.8.10-2011.7.21					
2007.4.25-2012.9.3	0	0	0	0	0
2006.9.6-2011.5.26					
2006.6.21-2009.6.1					
2006.1.22-2011.9.5					
2009.7.15-2011.10.26					1
2008.9.30-2010-12.16					
2008.11.26-2012.4.6	1				
2008.12.5	1	1	1		1
2006.8.4					
2007.11.14					
2006.5.8	1		1		1
2007.11.8	1	1	1		1
2006.10.24					
2007.6.18-2008.6.30					
2007.8.20					
2007.11.3					1
2007.11.28					
2008.1.5		1			
2008.2.1	0	0	0	0	0
2008.1.29-2008.6.23					
2009.7.16	1				1
2008.5.31	1		1		
2008.5.23					
2009.2.11					
2010.9.30-2011.4.14					1
2009.12.29					
2012.11					
2013.4					
2009.11.11					1
2009.2.11					
2009.5.13					1
2009.1.31		1			
2009.1.31	1				
2010.3.5		1			
2010.4.16	1	1			1
2010.4.20					
2009.12.28					
2009.6.5	1		1		1
2009.8.14			1		1
2010.1.27					
2010.5.25		1			
2010.7.14	0	0	0	0	0
2009.6.29					
2009.6.29初诊					
2009.9.11					
2010.12.3					
2009.2.24					
2011.10.27	0	0	0	0	0
2011.5.3起					
2012.4.6起	1	1	1	1	1

2012.5.18					
2007.4.9			1		1
2010.11.23					
2007.10.31				1	1
2011.8.17,9.2开始化疗					
2008.9.19开始治疗					
2012.6.29					
2011.11					
2014.4					
2009.2					
2013.3					
2012.9					
2012.9					
2012.4					
	1	1	1		1
2012.8					
2010.8					
2009.9.30	1	1			
2009.5.13		1			
2009.2.20					
2009.5.7					
2009.2.20					
2009.11.12	0	0	0	0	0
2009.4.17					
2009.6.22					1
2009.7.22					
2009.9.9			1		
2009.9.25	1	1			
2010.1.21					
2010.1.27					
2010.5.8	1				1
2010.4.6					
2010.7.8					
2010.4.2					
2010.6.25					
2010.6.9					
2010.12.8					
2010.9.25					
2010.10.11					
2010.12.16		1	1		1
2010.8.27					
2009.3.13					
2010.2.12	1	1			1
2010.4.26	0	0	0	0	0
2010.12.17		1			
2011.9.23					
2011.12.19	1	1			1
2011.2.23					
2011.3.23		1	1		1
2011.5.6					

2012.6.7					
2011.1.13					
2012.3.21	1	1	1		
2012.4.27					
2010.5.13					
2012.4.27					
2011.2.26					
2011.7.11					
2011.9.20					
2010.5.7	1	1			
2011.4.14					1
2011.2.18					
2011.9.20					
2011.11.1					
2012.4.20					
2011.11.9					
2012.3.30		1	1		
2011.7.11					
2011.4.27		1			1
2011.9.28					
2012.6.1					
2012.6.15					
11.6.22					

2013.3					
2013.4					
2013.2					
2012.12					
2012.10.11	0	0	0	0	0
2012.6			1		
2012.5					
2012.8					
2013.4		1	1		1
2012.12					1
2013.5					
2010.4.2		1			
2009.2.9					
2009.7.14		1			
2010.4.20					1
2009.12.11					
2010.5.5			1		1
2010.5.21					
2010.6.14					
2010.11.12					
2010.10.2					
2007.5.31		1			1
2010.7.5		1	1		

2009.12.18		0	0	0	0	1
2010.2.18		0	0	0	0	0
2010.5.8		1		1	0	1
2010.8.14						
2012.5.16						
2011.1.25	1					
2011.6.2		0	0	0	0	0
2011.5.27						
2011.1.18						
2011.10.22		0	0	0	0	0
2012.1.20						
2012.3.2			1			
2011.12.9	1		1			1
2010.11.8						
2011.10.28						
2012.2.22						
2011.8.5						
2011.6.8						
		1				
2010.3.5	0	0	0	0		0
2012.4	0	0	0	0		0
2012.8			1			
		1	1			1
		1				
2010.5						
2012.11						
2012.8	0	1	1	1		1
2012.8				1		1
2013.2						
2011.1						
2011.2						
2012.10.30		1				
2013.1		1				
2012.7						
2013.2	1	1	1	1		1
2011.11						
2013.4	0	0	0	0		0
2012.9						
			1			
2011.6.8						
2013.3	0	0	0	0		0
2013.4		1				
2012.6.20			1			
